# Supplementary material for: Splice-Junction-Based Mapping of Alternative Isoforms in the Human Proteome
Source: Cell Rep. Author manuscript; Available in PMC 2020 Jan 15. (PMC6961840; doi:10.1016/j.celrep.2019.11.026)
Supplement: 3 [file NIHMS1546469-supplement-3.zip › DF2/PXD0010154/Thyroid-145-P15692-SWSVPCGPCSER.pdf]

A

Predicted sequence disorder and sequence features of P15692

Peptide: SWSVPCGPCSER Junction: sp|P15692|VEGFA_HUMAN|ENSG00000112715|SE1|35337|chr6|43778918|43780803|+1|r95|T1 TrNovel: FALSE

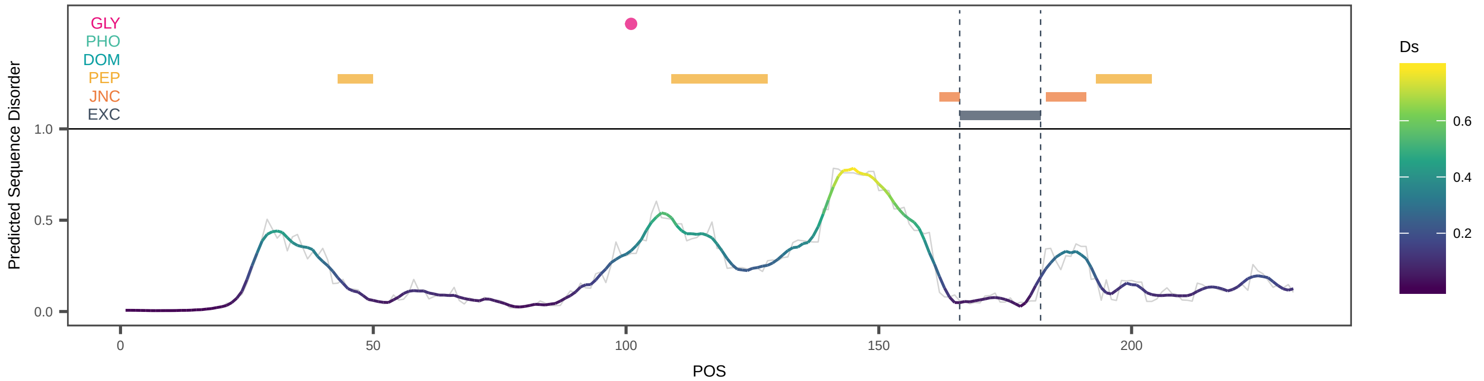

B

Distribution of sequence disorder in excised
vs. mapped and non−excised regions of protein

M−W P−value vs. mapped: 3.94e−09 vs. non−excised: 0.000205

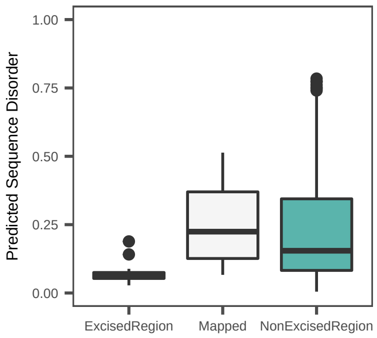

C
